# Dendritic Cells: Neglected Modulators of Peripheral Immune Responses and Neuroinflammation in Mood Disorders?

**DOI:** 10.3390/cells10040941

**Published:** 2021-04-19

**Authors:** Rafael Leite Dantas, Jana Freff, Oliver Ambrée, Eva C. Beins, Andreas J. Forstner, Udo Dannlowski, Bernhard T. Baune, Stefanie Scheu, Judith Alferink

**Affiliations:** 1 Department of Mental Health, University of Münster, 48149 Münster, Germany; rafael.leitedantas@ukmuenster.de (R.L.D.); Jana.Freff@ukmuenster.de (J.F.); udo.dannlowski@uni-muenster.de (U.D.); Bernhard.Baune@ukmuenster.de (B.T.B.); 2 Cells in Motion Interfaculty Centre, University of Münster, 48149 Münster, Germany; 3 Department of Behavioural Biology, University of Osnabrück, 49076 Osnabrück, Germany; oliver.ambree@uni-osnabrueck.de; 4 Center of Cellular Nanoanalytics, University of Osnabrück, 49076 Osnabrück, Germany; 5 Institute of Human Genetics, University of Bonn, School of Medicine & University Hospital Bonn, 53127 Bonn, Germany; e.beins@uni-bonn.de (E.C.B.); forstner@staff.uni-marburg.de (A.J.F.); 6 Institute of Neuroscience and Medicine (INM-1), Research Center Jülich, 52428 Jülich, Germany; 7 Department of Psychiatry, Melbourne Medical School, The University of Melbourne, Parkville, VIC 3010, Australia; 8 The Florey Institute of Neuroscience and Mental Health, The University of Melbourne, Parkville, VIC 3010, Australia; 9 Institute of Medical Microbiology and Hospital Hygiene, University of Düsseldorf, 40225 Düsseldorf, Germany; stefanie.scheu@uni-duesseldorf.de

**Keywords:** mood disorder, bipolar disorder, major depressive disorder, neuroinflammation, inflammation, dendritic cell, innate immune response

## Abstract

Affective disorders (AD) including major depressive disorder (MDD) and bipolar disorder (BD) are common mood disorders associated with increased disability and poor health outcomes. Altered immune responses characterized by increased serum levels of pro-inflammatory cytokines and neuroinflammation are common findings in patients with AD and in corresponding animal models. Dendritic cells (DCs) represent a heterogeneous population of myeloid cells that orchestrate innate and adaptive immune responses and self-tolerance. Upon sensing exogenous and endogenous danger signals, mature DCs secrete proinflammatory factors, acquire migratory and antigen presenting capacities and thus contribute to neuroinflammation in trauma, autoimmunity, and neurodegenerative diseases. However, little is known about the involvement of DCs in the pathogenesis of AD. In this review, we summarize the current knowledge on DCs in peripheral immune responses and neuroinflammation in MDD and BD. In addition, we consider the impact of DCs on neuroinflammation and behavior in animal models of AD. Finally, we will discuss therapeutic perspectives targeting DCs and their effector molecules in mood disorders.

## 1. Introduction

Affective disorders (AD) including major depressive disorder (MDD) and bipolar disorders (BD) are common mental disorders accompanied by enhanced morbidity, mortality, and suicidal risk. MDD is the most common AD, with an estimated lifetime prevalence of around 15% [1]. Core symptoms of a major depressive episode include depressed mood, decreased interest or pleasure (anhedonia) in almost all activities, and fatigue or loss of energy over the same two-week period. MDD often follows a chronic course with at least one-third of patients experiencing recurrent episodes within a year of stopping treatment [2]. BD is a severe and chronic recurrent mental disorder and is clinically characterized by extreme changes in mood, energy, and activity levels. It can be further divided into several subtypes, including Bipolar I (BD-I) and Bipolar II disorder (BD-II) [3]. BD-I is mainly characterized by mood swings between severe mania, which strongly interferes with daily functioning, and depression. The course of BD-II, on the other hand, is typically characterized by depressive and hypomanic states. Patients with BD suffer from poorer quality of life and suicide rates are 10–30 times higher than in the general population. The estimated lifetime prevalence of >1% in the global population is lower than that for MDD [4]. The etiologies of MDD and BD are multifactorial and not yet fully understood. An imbalance of neurotransmitters in the brain, dysfunction of the hypothalamic-pituitary-adrenal (HPA) axis, and neurodegenerative processes have been linked to the pathogenesis of AD. It is now known that a complex interplay of genetic and environmental factors contributes to the manifestation of AD including common and rare genetic variants, stress-induced epigenetic changes, and long-lasting effects of early life-trauma [4,5,6,7].

A plethora of findings suggests that the immune system plays a role in the pathophysiology of AD. For example, co-morbidities of MDD with autoimmunity and inflammatory disorders have been reported [8]. In addition, elevated levels of inflammatory markers have been found in depressed individuals and in rodents with depression-like behavior, as well as in response to stressful events [9,10,11]. In accordance, individuals with AD often have elevated serum levels of pro-inflammatory cytokines, e.g., tumor necrosis factor (TNF), interleukin (IL)-1β, and IL-6 [12]. Findings in this area led to the formulation of the “inflammation hypothesis of depression” almost 30 years ago. Maes and colleagues observed that depression is associated with a mild “inflammation” characterized by monocyte and T cell activation and increased levels of circulating inflammatory factors [13]. The link between the immune system and depression was supported by reports showing that depressive symptoms occurred in patients after immunotherapy with type I interferons (IFN) [14,15]. Since then, these authors and others have updated the hypothesis to include pathophysiological mechanisms such as oxidative stress, neurodegenerative processes, and altered neurogenesis in MDD [16,17,18]. The immune hypothesis is further strengthened by meta-analyses showing increased levels of C-reactive protein, the cytokines IL-6, IL-12, IL-18, and TNF, and the chemokine CCL2 in depressed individuals [8,19,20,21]. Mechanistically, cytokines in the blood can enter the CNS and decrease monoamine levels, increase microglia activation and oxidative stress, processes that have been linked to cognitive deficits and mood changes [22,23]. Furthermore, polymorphisms in genes encoding inflammatory cytokines, including IFN-γ and IL-18, have been associated with dysregulated amygdala reactivity to emotional stimuli and MDD following a history of stressful life events [24,25]. Early-life stress such as childhood maltreatment is the strongest environmental risk factor for AD and has been associated with long-term immune changes and increased susceptibility to MDD [26,27,28]. Moreover, clinical trials involving chronic inflammatory conditions showed that anti-inflammatory treatments mediated antidepressant effects [29,30,31,32]. Finally, depression-like “sickness behavior” in rodents after treatment with inflammatory mediators underscored the bidirectional relationship between depression and immune processes [33].

Dysregulation of the immune system has also been implicated in the pathophysiology of BD. Here, an immune hypothesis was first formulated by Horrobin & Lieb in 1981 [34]. The authors postulated that the mood-stabilizing effect of lithium is mediated by suppression of T cell function during manic episodes and enhancement of T cell activities during depressive episodes. Epidemiological studies also indicated enhanced comorbidity of inflammatory diseases such as autoimmunity, chronic infections, and metabolic disorders with BD [22]. Other studies linked alterations in cytokine/chemokine serum levels to mood states, although specific patterns of inflammatory markers that distinguish MDD from BD remain controversial [22,35,36,37].

Regarding the cellular components involved in the interplay between AD and the immune system, many previous studies have focused on monocytes, macrophages and/or microglia [38,39,40,41,42,43,44,45,46]. Only recently, few studies have shown a possible influence of dendritic cells (DCs) and their effector molecules in AD and disease-associated behaviors. DCs are professional antigen presenting cells that provide the link between the innate and adaptive immune system. Together with monocytes/macrophages, granulocytes, and natural killer (NK) cells, they constitute the first line of defense against invading microbial pathogens. DCs also play a central role as mediators of tolerance in peripheral immune responses and neuroinflammation [47,48]. The aim of this review is to shed light on the current knowledge of DCs in the pathophysiology of AD. To this end, clinical studies reporting phenotypic changes of DCs and their effector functions in individuals with depressive symptoms or diagnosed MDD and BD are reviewed. In addition, recent findings demonstrating a functional role of DCs in rodent behavior and, conversely, the effects of chronic stress exposure as a risk factor of AD on basic DC functions will be highlighted. Finally, the potential of targeting DCs as a future treatment option of AD will be discussed.

## 2. Selected Functions of DCs with Relevance for Mood Disorders

DCs were originally discovered in 1973 by Steinman and Cohn and named after their stellate or “dendritic” morphology exhibiting tree-like veils [49,50]. DCs represent a heterogeneous group of bone marrow-derived cells that are widely distributed throughout the body. Their diverse morphology, phenotype, and function depends on their origin, the transcriptional control of their development, and the respective microenvironment [47,51,52,53,54]. DCs are found in lymphoid tissues such as bone-marrow, thymus, spleen, lymph nodes, and Peyer’s patches, as well as in non-lymphoid tissues and in peripheral blood [47,51,52]. In an immature or semimature state, DCs screen peripheral tissues for “danger signals” using pattern-recognition receptors (PRRs). PRRs represent germline-encoded receptors in the cytosol or membrane compartments of immune cells. They sense evolutionarily conserved structures from pathogens termed pathogen-associated molecular patterns (PAMPs). PAMP binding to PRRs on DCs and other innate immune cells induces a cascade of effector mechanisms involving phagocytosis and production of inflammatory factors, reactive oxygen species, and nitric oxide to eliminate the danger [55,56]. One of these PAMPs is lipopolysaccharide (LPS), the major membrane glycolipid of Gram-negative bacteria, that binds toll-like receptor (TLR) 4. Experimental administration of LPS is commonly used in humans and rodents to induce “sickness behavior” associated with emotional and inflammatory changes, such as depressed mood and altered serum levels of proinflammatory cytokines [57,58,59]. LPS also affects the phenotype and function of DCs. It induces maturation of DCs, which in this process upregulate major histocompatibility complex (MHC) and costimulatory molecules and secrete cytokines and chemokines to induce naïve T cell activation. DCs express a variety of PRRs, including TLRs, NOD-like receptors (NLRs), retinoic acid-inducible gene I (RIG- I)-like receptors (RLRs), purinergic receptors, C- type lectin receptors (CLRs), and the receptor for advanced glycation end products (RAGE) [60,61,62]. They can thus sense a broad range of danger signals and quickly respond to pathological conditions and stressors in their surroundings.

Damage-associated molecular-patterns (DAMPs), on the other hand, are released from stressed or dying cells upon stress or tissue injury and are also sensed by PRRs. DAMPs trigger “sterile inflammation” even in the absence of infection [61]. Specific DAMPs such as S100 proteins, high-mobility group box 1 (HMGB1), heat shock proteins (HSPs), and ATP have been associated with the pathogenesis of mood disorders and are involved in stress-induced depression-like behaviors in mice [63,64]. PAMP and DAMP binding to PRRs of the NLR family, activates the pyrin domain-containing 3 (NLRP3) inflammasome complex leading to caspase-1 activation and maturation of IL-1β. The NLRP3 inflammasome has been shown to bridge stress-induced sterile inflammation and depression [65,66].

Innate immune responses mediated by activation of germline-encoded PRRs by PAMPs or DAMPs are not antigen-specific in contrast to the activation of T and B lymphocytes in adaptive immune responses. Moreover, the development of long-term immunological memory was considered to be an exclusive capacity of the lymphocytes of the adaptive immune system. This dogma has recently been challenged by studies showing that innate immune cells, including DCs and macrophages, can provide an adjusted immune response after the first encounter with a pathogen. This so called “trained immunity” is mediated by long-term functional reprogramming of innate immune cells through metabolic and epigenetic adaptations [67,68]. Similarly, innate immune cells may also exhibit long-term adaptations following early-life stress and/or chronic stress, both risk factors for AD that lead to glucocorticoid resistance and chronic production of inflammatory cytokines [16,69]. After encountering the pathogen, DCs undergo phenotypic maturation and upregulation of chemokine receptors required for migration to regional lymph nodes. To initiate the adaptive immune response, DCs present exogenously derived antigenic peptides mostly to naïve CD4^+^ T cells via MHC II, whereas endogenous peptides bound to MHC I stimulate naive CD8^+^ T cells [47]. In a process termed cross-presentation, DCs also present exogenous antigen on MHC I to CD8^+^ T cells for elimination of virus infected or tumor cells [70,71]. Compared to other APCs such as B cells and macrophages, mature DCs express by far the largest amount of MHC II on their cell surface [72].

DCs are able to secrete a variety of pro-inflammatory cytokines such as IL-1β, IL-6, IL-12, IL-23, and TNF that are involved in the pathophysiology of mood disorders. For example, IL-12 production by DCs induces T helper (Th)1 responses and natural killer (NK) cell activation, processes involved in both the defense against intracellular pathogens and the pathophysiology of depression. On the other hand, production of IL-6 and IL-23 by DCs promotes pathogenic Th17 responses that trigger autoimmunity and depression-associated behaviors. Conversely, DCs also promote immune tolerance through secretion of anti-inflammatory factors (e.g., IL-10 and transforming growth factor (TGF)β), expansion of regulatory T cells (Tregs), and upregulation of the immunosuppressive enzyme indoleamine 2,3-dioxygenase (IDO), a rate-limiting enzyme in tryptophan metabolism [47,73]. All of these immune processes are frequently altered in MDD patients and depression-like behavior. For example, IL-10 knockout mice show increased depression-like behavior and administration of IL-10 rescued depression-associated learning and memory deficits in mice [74,75]. In addition, Treg insufficiency has been found in patients with MDD. Grosse et al. (2016) showed that in MDD the percentage of circulating Tregs was inversely associated with the activation state of monocytes, which are precursors to DCs and macrophages [76]. Finally, increased IDO activity was found in LPS induced depression-like behavior [77]. Thus, overall, a large number of findings point toward the involvement of DCs in mood disorders.

## 3. Human and Mouse DC Subsets

DCs in human and mouse are commonly classified on the basis of their phenotype and function into three major subsets, namely conventional DCs (cDCs), plasmacytoid DCs (pDCs), and monocyte-derived DCs (moDCs) (Table 1) [78,79]. As shown in Table 1, major DC subsets are characterized by different sets of surface and intracellular markers (Table 1). On a functional level, cDCs are mainly specialized in presenting exogenous or endogenous antigens to naïve T cells [79]. In contrast, pDCs harbor the capacity to rapidly produce large amounts of type I IFN during antiviral immune responses [80,81]. moDCs arise from monocyte precursors, especially during inflammatory processes when their number can increase rapidly [79]. Like cDCs, they are mainly involved in inducing T cell responses [82]. While all three DC subsets can be differentiated and cultured from mouse bone-marrow derived DCs, moDCs can also be generated in vitro from human blood monocytes [83]. These are therefore one of the best studied DC subsets in the context of mood disorders.

### 3.1. Plasmacytoid DCs

pDCs represent a small subset of DCs that comprise approximately 0.1% to 0.5% of nucleated cells in lymphoid organs. Unlike other DCs subsets, pDCs have a plasma cell-like morphology [80]. In humans, they can be found in lymphoid tissues, lung, and peripheral blood [79]. Exogenously administered granulocyte-macrophage colony-stimulating factor (GM-CSF), which has been shown to ameliorate LPS-induced depressive symptoms in mice [84], inhibits pDC development (via STAT5) by regulating the expression of its transcription factor basic helix-loop-helix protein E2-2 (E2-2 also known as TCF4) [79,85]. In addition, the transcription factors interferon regulatory factor 8 (IRF8), B-cell lymphoma/leukemia 11A (BCL11A), and PU.1 are required for their development [86,87]. pDCs sense viral RNA by TLR7 and CpG containing DNA by TLR9 ligation [81,88]. pDCs were first described as the main producer of type I IFN in human blood [89,90]. The rapid type I IFN production capacity of pDCs is based on unique molecular adaptations to nucleic acid sensing [81]. Meanwhile, findings from type I IFN reporter mouse lines have shown that only a small subset of pDCs activates type I IFN expression in in vivo infection models [81]. These type I IFN-producing pDCs have been shown to exhibit a distinct dynamic gene expression profile that is tightly controlled in time and localization of the pDC within the microarchitecture of lymphoid organs. This leads to a highly coordinated expression of cytokines, chemokines, and costimulatory molecules by these pDCs facilitating T cell recruitment and activation [91,92]. In addition to type I IFN, pDCs can also produce type III IFN, and other cytokines such as IL-6, IL-8, IL-12p40, and TNF, as well as various chemokines [93].

### 3.2. Conventional DCs

cDC stem from a common DC progenitor (CDP) and are located in virtually all lymphoid and non-lymphoid tissues [94,95]. Human cDCs have been subdivided into two subsets (cDC1 and cDC2). The development of each subset is based on differential expression of key transcription factors. While cDC1 development depends on IRF8, inhibitor of DNA binding 2 (ID2), and basic leucine zipper transcriptional factor ATF-like 3 (BATF3), cDC2 development is driven by IRF4, Neurogenic locus notch homolog protein 2 (Notch2), and Kruppel-like factor 4 (KLF4). cDC1 are involved in the induction of type 1 immune responses and the differentiation and activation of group 1 innate lymphoid cells (ILC1), NK, and Th1 cells [96]. Secretion of IL-12 is a major mechanism by which cDC1 cells mediate their functions [97]. cDC1s are also capable to cross-present extracellular antigens to CD8^+^ T cells and deletion of BATF3 abolished the development of cDC1s in mice along with cross-presentation [98,99]. cDC1 are also able to upregulate expression of the immunosuppressive enzyme IDO, which has been shown to be involved in the pathogenesis of depression, especially in a context of high IFNα levels [16,100]. Accordingly, treatment of hepatitis C patients with IFNα induced depressive symptoms. Psychopathological symptoms were associated with increased IDO activity measured indirectly by quantifying kynurenine, a neurotoxic metabolite produced by IDO [100]. cDC1 may further induce Treg cell-mediated immune tolerance, a process that seems to be dependent on antigen presentation and is organ-specific [101].

cDC2 in humans are approximately 10 times more frequent than cDC1 under steady-state conditions. cDC2s are highly proficient in MHC II-mediated antigen-presentation to CD4^+^ T cells and promote polarization of Th2 cells against multicellular parasites as well as in allergic diseases [102]. When activated, cDC2s secrete IL-1β, IL-6, IL-10, IL-12, IL-23, and TNF [79,103]. By secreting IL-6 and IL-23, both cytokines relevant for Th17 cell differentiation and maintenance, they regulate the balance of Tregs and Th17 cells [104]. Imbalance between Tregs and Th17 cells is related to autoimmune diseases and MDD [105,106].

**Table 1:** Human and mouse DCs subsets.

DC Subset	Transcription Factors	Major Cytokines	Major Surface Makers		Major PRRs		Reference
			Human	Mouse	Human	Mouse	
pDCs	IRF8, BCL11A, E2-2/TCF4	type I IFN	CD123/IL-3RA, CD303/CLEC4C/BDCA-2, CD304/NRP1/BDCA-4 and HLADR ^low^	CD11c ^low^, B220, CD317, Siglec-H, CD172a, CD209, CCR2 ^low^, CCR9, CXCR3 and MHC II ^low^	TLR7 and TLR9	TLR7 and TLR9	[79,83,96]
cDC1s	BATF3, IRF8, ID2, Zbtb46 (BTBD4)	IL-12	CD11c ^low^, HLA-DR, CD141/BDCA1, XCR1, CLEC9A/DNGR1, DEC205, IDO	CD11c, MHC II, CD8α (resident), CD103 (migratory), CD24, XCR1, CLEC9A and DEC205	TLR3 or CLEC12A	TLR4 or CLEC12A	[79,96,107,108]
cDC2s	ID2, Zeb2, NOTCH2, IRF4, KLF4, Zbtb46 (BTBD4)	IL-1β, IL-6, IL-10, IL-12, IL-23, and TNF	CD1c/BDCA-1, CD2, CD172a/SIRPA, CD11c, HLA-DR, CD11b, CD1a (migratory), FcεR1, ILT1, CD14 and CD5 (subset)	CD11c, MHC II, CD11b ^high^, CD172a/SIRPA	TLRs 1-9	TLRs 1-9	[79,96]
moDCs	CSF1R, MAFB, KLF4, Zbtb46 (BTBD4)	IL-1β, IL-6, IL12, IL-23, and TNF	CD11c, HLA-DR, CD1c, CD11b, CD14, CD64, CD206, CD209, CD172a, CD1a, CCR2	CD11c, MHC II, CD11b, Ly6C, CD64, CD206, CD209, CD14, CCR2	-	-	[109,110]

Adapted from Wculek and co-authors [96].

### 3.3. Monocyte-Derived DCs

moDCs or so called inflammatory DCs (infDCs) are a subset of DCs that differentiate from monocytes during inflammatory conditions in vivo [79]. moDCs can also be generated in vitro from human blood monocytes or from murine bone marrow (e.g., by cultivation in the presence of GM-CSF and IL-4) [83]. moDC development depends on the transcription factors known from cDC1s, such as BATF3, and from cDC2s, like IRF4. In the absence of IRF4, GM-CSF and IL-4-stimulated monocytes differentiate into macrophages instead of moDCs, showing that moDC, like cDC2, are dependent on IRF4 [111]. moDCs may exhibit regulatory functions in steady state human tissue, like the lungs, intestine, and skin. In addition, they are involved in the generation of Tregs [96]. Finally, human moDCs can secrete various cytokines, such as IL-1β, IL-6, IL12, IL-23, and TNF. Like cDC2, they are potent inducers of Th17 polarization by secretion of IL-6 and IL-23. In addition, they are involved in Th1, Th2, and CD8^+^ T cell expansion [109]. moDCs, therefore, can be regarded as a very complex cDC subset being able to take over many functions from cDC1 and cDC2.

## 4. DCs in Mood Disorders and Depression-Like Behavior

### 4.1. Genetic Studies with a Relation to DCs in Mood Disorders

Mood disorders are multifactorial disorders with genetic and environmental factors contributing to their development. Based on twin and family studies, the heritability of MDD and BD is estimated to be around 40% and 60–85%, respectively [112]. GWAS and GWAS meta-analyses have provided the most comprehensive insights into the genetic basis of MDD and BD to date. 

Recently, Howard and colleagues performed a GWAS meta-analysis of depression and investigated data from around 246 K cases and 561 K controls [113]. In this study, 102 independent genome-wide significant variants in 101 genomic loci were identified. The associated loci included the extended MHC region on chromosome 6, which harbors numerous immune-related, but also non-immunological genes [114,115]. A study by Glanville and colleagues (2020), however, did not provide evidence that the association with depression is driven by variation in the classical HLA alleles [116]. Pathway analysis of the major depression GWAS data by Wray et al. (2018) revealed significant enrichment for genes involved in the regulation of cytokine production involved in the immune response [117].

The currently largest GWAS of BD investigated ~42 K BD cases and ~371 K controls and identified 64 genome-wide associated loci [118]. As in depression, these loci included the extended MHC region. Pathway analysis of the GWAS data, however, did not reveal significantly enriched immune-related gene sets [118]. Using GWAS and expression data as well as innovative biostatistical methods a significant enrichment for specific blood/immune cell types was found for BD, i.e., neutrophils, hematopoietic stem cells, and leukocytes [119,120]. However, significant enrichment for DCs was not found for either BD or depressive symptoms after correction for multiple testing [120,121]. While these findings do not provide strong evidence for DCs as disease-relevant cell type in mood disorders, individual associated genomic loci may nevertheless mediate their contribution to disease development via altered DC function. The detailed mechanisms and causal genes are still unknown for most genome-wide significant loci and have yet to be elucidated in future functional studies. However, closer inspection of genes located at genome-wide significant loci may provide initial clues to a potential involvement of DCs.

Interestingly, GWAS of MDD and BD identified risk loci harboring genes that regulate DC function and development. It should be noted, however, that these genes are typically not exclusively expressed by DCs and that the identified disease associations could thus be mediated through other cell types or mechanisms. The genome-wide significant risk variants reported by Wray et al. (2018) include the single-nucleotide polymorphism (SNP) rs12958048, which is located in the *TCF4* gene encoding the transcription factor E2-2/TCF4 [117]. *TCF4* has previously been reported as a genome-wide significant risk locus for schizophrenia [122]. As mentioned above, TCF4 is the master regulator of pDC development in humans and mice, and their capacity to produce type I IFN [123]. Type I IFN is known to induce depressive symptoms in humans, suggesting an influence of pDCs as specialized type I IFN producers in inflammatory responses [14,15]. 

The currently largest GWAS of BD identified a genome-wide significant variant in the *CACNA1C* gene on chromosome 12 [118]. Besides BD, variants at the *CACNA1C* locus have also been associated with other psychiatric disorders including schizophrenia [122]. *CACNA1C* encodes the pore forming subunit of the voltage-dependent L-type gate calcium ion (Ca^2+^) channel (LTCC) Cav1.2 that regulates depolarization-dependent Ca^2+^ influx into cells. CACNA1C plays important roles in neuronal functions and survival and synaptic plasticity, and therefore modulates learning and memory behavior [124]. Interestingly, the *CACNA1C* gene is not only widely expressed in the nervous system, but also found in immune cells such as DCs. Multiple functions of DCs such as maturation, migration, and immunological synapse formation with T cells depend on Ca^2+^ signaling. Cav1.2 is directly involved in antigen presentation of DCs as it has been shown to activate Ryanodine Receptor-1 (RyR1) signaling causing rapid MHC II expression in the membrane of DCs [125].

Overall, there is currently no strong evidence for the direct involvement of AD-associated genetic variants in regulating DC function, as most implicated risk genes are expressed in various cell types. Future genetic and functional studies are needed to assess the contribution of DCs to the development of MDD and BD, which, if present, may only refer to specific etiological patient subgroups. In addition, only specific DC subsets or individual differences in immune responses might be relevant for disease pathogenesis. Therefore, more refined integrative analyses of genetic data with data from state-of-the-art sequencing methods (e.g., single-cell RNA sequencing) and/or immune response expression and expression quantitative trait loci (eQTL) studies in DCs should be conducted in the future [126,127].

### 4.2. DC-Expressed Chemokines and Chemokine Receptors Involved in Mood Disorders and Depression-Like Behavior

Migration of DCs in homeostasis and inflammation is orchestrated by chemokine/chemokine receptor interactions. Chemokines form a large family of small chemotactic proteins that control leukocyte trafficking and cellular processes such as cell adhesion, activation and proliferation, and cytokine secretion through signaling via G protein-coupled receptors [128,129]. Chemokines also control proliferation and migration of neural precursor cells or mature neurons, as well as glial cells and are therefore involved in CNS development and homeostasis [130,131]. Several chemokines and their receptors modulate stress responses, and increased levels of chemokines have been found in depressed individuals and depression-like behavior [132]. In the following chapter, we will focus on selected chemokine/receptors involved in DC biology and at the same time in mood disorders.

The chemokine receptor CCR7 is the cognate receptor for the ligands CCL19 and CCL21 and plays a crucial role in the organization and homeostasis of the microarchitecture of lymphoid organs (Table 2). Upon maturation, CCR7 is upregulated on DCs and controls their migration from peripheral tissues to regional lymph nodes and thus provides a link between peripheral inflammatory processes and neuroinflammation [133]. CCR7 binding to any of its ligands increases the endocytosis capacity, differentiation, and cytokine production ability of DCs [134]. Deficiency of CCR7 in mice confers resistance to CNS autoimmunity due to a defect of DCs to produce IL-23 and IL-12 and to induce pathogenic Th17 cells [135]. CCR7 was also shown to affect cognition and emotional behavior. CCR7^-/-^ mice exhibited learning and memory deficits and higher levels of anxiety than WT animals. In addition, CCR7^-/-^ mice showed decreased preference for saccharin in weekly testing indicating that CCR7 modulates depression-like behavior [136].

Many chemokine receptors, including CCR1, CCR2, CCR5, CCR6, CXCR1, and CXCR2 have been shown to direct chemotaxis of immature DCs to sites of inflammation [143]. For example, CCR2 expressed by DCs drives their maturation, migration, and IL-12 production via activation of the transcription factor NF-κB. Clinical studies reported higher serum concentrations of its ligand, CCL2, in depressed individuals as compared to healthy controls, and vice versa, antidepressants have been shown to reduce CCL2 levels [30,132]. Elevated CCL2 serum levels have also been found in patients with BD [140] suggesting an impact of the CCR2/CCL2 axis in mood disorders. So far, the biological function of CCR2-expressing monocytes has been in the focus of studies regarding mood disorders. The influence of this chemokine/receptor pair in DCs in the pathophysiology of mood disorders needs to be addressed in future studies.

CXCL8 (IL-8), as well as other chemokine ligands (CXCL1, CXCL2, CXCL3, CXCL5, CXCL6, CXCL7) act on CXCR1 and CXCR2 receptors. CXCL8 is known to primarily induce chemotaxis of CXCR1- and CXCR2-expressing neutrophils to inflammatory sites. CXCL8 also mediates its biological effects on immature DCs that express the cognate receptors [144]. A comprehensive meta-analysis showed increased CXCL8 levels in the blood of depressed individuals compared to controls [141]. However, it is unclear whether the observed chemokine changes are primary or secondary to MDD and whether enhanced levels of CXCR1 and CXCR2 ligands affect actions of DCs in mood disorders.

In marked contrast to most other chemokine receptors, CCR6 has only one known ligand, namely CCL20. The CCL20/CCR6 axis controls chemotaxis of DCs to inflammatory sites and the brain and has been implicated in chronic inflammatory conditions (e.g., inflammatory bowel disease, chronic liver disease), neuroinflammation and neurodegeneration [145,146]. Direct evidence of CCR6 involvement in depression-like behavior came from CCR6^−/−^ mice showing an anhedonic phenotype as indicated by reduced preference for saccharin compared to WT animals [136].

CXCR4/CXCL12 engagement is essential for migration of cutaneous DCs into the regional lymph nodes [147] and mediates retention of DC precursors in the bone marrow of mice [148]. In addition to these immune functions, CXCL12 has also been shown to play important roles in the CNS. CXCL12 is expressed in the brain by glial cells and neurons and controls axonal guidance and neurite outgrowth [149,150]. Plasma levels of CXCL12 have been found reduced in patients with non-affective psychosis compared to healthy controls [142]. Moreover, CXCL12 increases the synaptic activity of gamma-aminobutyric acid (GABA) and glutamate at serotonergic neurons in the rat dorsal raphe nucleus and the proliferation of human neural progenitor cells in vitro. Both neurotransmitters, GABA and glutamate, are involved in the pathophysiology of mood disorders [151,152,153].

CX3CR1 represents another chemokine receptor that is expressed by DCs during all stages of their differentiation. CX3CR1 modulates DC trafficking mainly through inflamed lymphatics. CX3CR1 is also found on other immune cells, such as monocytes and microglia in the CNS. CX3CR1 is the receptor for the only known member of the CX3C chemokine family, CX3CL1 (Fractalkine). Fractalkine is expressed in neurons, intestinal epithelium, and activated lymphatic endothelial cells [154]. Absence of CX3CR1 exacerbates LPS-induced neuroinflammation in mice but increases resilience to stress-induced depression-like behaviors [138,155]. Resilience of CX3CR1 deficient mice in a chronic despair model was mainly attributed to altered neuron-microglia signaling via CX3CR1/CX3CL1 due to hyperbranched microglia. A positive correlation between CX3CL1 levels and depression severity was observed in patients with colorectal cancers and comorbid depression. The CX3CR1/CX3CL1 axis is also discussed as a target for the treatment of many chronic inflammatory diseases, including Alzheimer’s disease, atherosclerosis, and asthma [154]. CX3CL1 effects involving DCs in mood disorders have not been studied so far.

CCR4 is the cognate receptor for CCL17 and CCL22, which play important roles in DC-mediated peripheral inflammatory responses and neuroinflammation. CCR4 is expressed by DCs and other immune cells, including T cells, NK cells, and macrophages/monocytes [48,156,157]. We have shown before that DCs are the primary cellular source of CCL17 using an Enhanced Green Fluorescent Protein (EGFP) expressing reporter mouse model [158]. We also demonstrated that CCR4 and CCL17 are functionally involved in CNS autoimmunity by regulating DC functions [159,160]. CCL17-deficient mice show reduced clinical severity of experimental autoimmune encephalomyelitis (EAE) due to a defect of peripheral DCs to migrate into the CNS [160]. In the brain, CCL17 is expressed in hippocampal neurons upon inflammatory stimulus and CCL17 deficiency confers phenotypic alterations in microglia such as reduced cellular volume and a more polarized process tree compared to WT controls [161]. In the absence of CCR4, mice are resistant to the development of EAE. Mechanistically, CCR4-deficient DCs are less able to secrete GM-CSF and IL-23 in the CNS and to promote the survival of pathogenic Th17 cells [159]. CCR4-deficient mice also exhibit behavioral changes such as reduced locomotor activity, less anxiety-related behavior, and diminished social exploration compared to WT animals [137]. In contrast, CCL17 deficient mice showed no altered behavior suggesting a mechanistic or developmental role of CCR4 in the regulation of these behaviors. These findings in sum demonstrate that the CCL17/CCL22/CCR4 axis is an essential modulator of neuroinflammation and behavior suggesting a potential role in inflammation-induced depression. In accordance, findings in humans showed higher CCL22 blood levels in patients with MDD who responded to anti-depressive therapy [162].

### 4.3. DC-Derived Cytokines and Chemokines and Their Potential Influence on Microglia Function

DC-dependent peripheral immune responses are closely associated with neuroinflammation and microglia activation [159,160,163]. Microglia are immune effector cells of the brain and fulfill numerous functions in neurodevelopment, neuroprotection, and immunosurveillance. Microglial dysfunction has been found in many inflammatory, autoimmune, and neurodegenerative CNS disorders, including mental disorders. Postmortem and imaging studies in humans, as well as neuroimmunological analyses in rodent models reported microglial activation in MDD and depression-associated behavior [40,44]. Microglia express PRRs and a plethora of additional immune receptors, including those for cytokines and chemokines. Upon encounter of an inflammatory stimulus or pathogen, they develop an amoeboid phenotype, express higher levels of MHC and co-stimulatory molecules, acquire migratory competence, and release inflammatory cytokines/chemokines that amplify the inflammatory response [164,165,166]. Moreover, peripheral innate immune challenge resulting in enhancement of inflammatory cytokines has been shown to induce microglia activation. As described above, peripheral DCs and monocytes/macrophages are a prominent cellular source of Type I IFNs, IL-1β, IL-6, and TNF that reach the CNS via the humoral pathway [167]. It is therefore likely that DCs orchestrate microglial activation and increase their migratory and phagocytic capabilities during neuroinflammation in AD.

Microglia acquire multiple phenotypes associated with distinct molecular signatures. However, the microglial phenotype associated with MDD and depressive-like behavior is still a matter of debate. Many studies point to the predominance of classically activated, pro-inflammatory M1 microglia in stress responses and major depression. For example, positron emission tomography (PET) using PET ligands such as the microglia marker translocator protein 18 kDa (TSPO) showed microglia activation in major depression and correlated TSPO expression levels with severity and duration of illness [168]. Social defeat in rodents used as stress/depression model induced microglial activation and increased expression of pro-inflammatory cytokines in brain regions associated with fear and anxiety [169]. M1 microglia are induced by PRR ligands, IFNγ, and GM-CSF. We have shown before, that CCR4^+^ DCs capable to invade the brain during neuroinflammation in a model of CNS autoimmunity are specialized to produce GM-CSF [159]. On the other side, lack of microglial activation or an immune-suppressed microglial state has been found in depressed individuals (for review see Yirmiya, Rimmerman and Reshef, 2015) [170,171]. Recent findings using single cell mass cytometry of microglia isolated from postmortem tissues of individuals with MDD support the view of a homeostatic, but not inflammatory marker profile of these cells [172]. Among other cells, DCs are also capable to release anti-inflammatory IL-10 and TGFβ and may thereby support alternative activation of M2 microglia specialized to mediate tissue repair, immune regulation, and/or phagocytosis [165,173,174]. Thus, although many immune factors produced by DCs directly affect microglial functions, we do not have yet a coherent picture of how DCs are involved in these processes in AD. Further studies are therefore needed to better understand the impact of DCs in microglial polarization in the pathophysiology of AD.

### 4.4. DCs as Modulators of Adaptive Immune Responses in Mood Disorders

DCs connect innate and adaptive immune responses through PAMP/DAMP recognition on the one hand and their ability to induce activation of naïve T cells on the other. After antigen recognition within a specific cytokine milieu, activated CD4^+^ T cells differentiate into a variety of effector Th cell subsets, including Th1, Th2, Th17 and Treg cells (Tregs) [175,176]. Th1 cells produce, among others, the lead cytokines IL-2 and IFNγ and play an important role in the clearance of intracellular pathogens. Their development is favored by IL-12. The presence of IL-4 promotes the development of Th2 cells that are involved in immune responses against extracellular pathogens and antibody class switching. Th17 cells play a pathogenic role in the development of autoimmunity and inflammatory disorders [104]. TGFβ and IL-6 induce differentiation of Th17 cells, while IL-23 supports their maintenance. Tregs represent cellular counterparts of Th17 cells and due to their immunosuppressive capacities are involved in the development and maintenance of tolerance [177]. A plethora of studies have suggested a role for Th1, Th17 and Treg cells in the pathophysiology of MDD and altered Th17 and Treg cell numbers and functions have been found in stress-induced behavior in mice [76,178,179,180,181,182,183,184,185,186,187]. As there are excellent review articles on this topic, we may refer to previous publications [106,179,188,189].

Differentiation of naïve CD4^+^ T cells effector Th cell subsets is mainly controlled by DCs [97,190,191]. DC subtypes carry out distinct functions that shape Th cell differentiation and responses. For example, cDCs are able to produce IL-6, IL-12, and IL-23, thereby affecting the balance between Th1, Th2, Th17, and Treg cells [53]. By releasing IL-12, cDC1 cells promote Th1 responses. However, cDC1 have also been shown to induce Th1 differentiation in the absence of IL-12, and cDC2 cells promoted Th2 differentiation in the absence of IL-4. Thus, Th cell differentiation may be influenced by the specificity of the DC subset rather than the cytokines released [192]. Moreover, differences in the capacity of DC subsets to process and present antigens also affect T cell responses. Human pDCs can also induce the development of Tregs from naive CD4^+^ T cells by expression of IDO and programmed death-ligand 1 (PD-L1) [193,194]. Furthermore, DCs modulate Th cell responses via the secretion of MHC I and II containing exosomes, also known as extracellular vesicles (EV) [195,196]. Depending on the individual size of the EVs, they show different potential to promote Th1 versus Th2 cell responses. Immature DCs producing large EVs induce the secretion of Th2-associated cytokines, whereas small and medium EVs induce the secretion of Th1-associated cytokines in T cells. Despite the fact that DCs are master regulators of Th cell responses by using different strategies, studies unraveling the role of DCs in CD4^+^ Th cell regulation in mood disorders are still lacking. Future studies are needed to unravel their specific roles in driving different CD4^+^ T cell subsets in the pathophysiology of mood disorders.

### 4.5. Effects of Antidepressant Treatment on Human and Murine DCs

Several treatment studies for AD in humans indicate that DC phenotype and function may be affected by psychopharmacological treatments for AD. A pilot study compared vilazodone, a 5-HT1A receptor agonist/serotonin transporter inhibitor, with paroxetine for antidepressant and immunomodulatory effects in late-life depression [197]. The authors examined leukocyte gene expression profiles for specific proinflammatory gene transcripts. Both treatments equally improved depressed mood, but only vilazodone-treated samples exhibited relative reductions in many cardinal genes encoding pro-inflammatory cytokines, HLA-DR, and the costimulatory molecule CD83. Transcript origin analyses revealed that DCs and monocytes were the primary cellular source of down-regulated mRNAs in the vilazodone-treated group. Several of those encoded proteins are involved in antigen presentation and CD4^+^ T cell activation by DCs, such as HLA-DRB5, HLA-DRB1, CD83, and TNFAIP3 [197]. HLA-DRB5 and HLA-DRB1 polymorphisms are associated with neuroinflammatory and neurodegenerative diseases, such as multiple sclerosis, Alzheimer’s and Parkinson’s disease [198,199,200]. Analysis of HLA-DRB5 gene expression in peripheral blood might serve as a remission predictor for antidepressant treatment in late-life depression [201]. In addition, an association between HLA-DRB1 and post-traumatic stress disorder has been reported [202]. It can also be used as predictor of brain and cerebellar atrophy in patients with Gulf War Illness (GWI), a disease of veterans of the 1991 Gulf War [203]. The gene *TNFAIP3* (Tumor necrosis factor alpha-induced protein 3) encodes the zinc finger protein A20, an ubiquitin-modifying enzyme, that is also involved in DC functions. TNFAIP3 deficiency in DCs is known to result in higher Th17 differentiation capacity through increased expression of IL-1β, IL-6, and IL-23, and to inhibit the differentiation of Th2 cells by increasing levels of IL-12 and IL-6 [204]. In sum, vilazodone-induced mood improvement was linked to the downregulation of immune genes in DCs, which are related to DC maturation and T cell activation.

Two studies investigated the treatment effects of the mood stabilizer lithium on moDCs in BD. Wu and coauthors demonstrated that moDCs generated from lithium-treated patients with BD-I expressed higher levels of CD14, a co-receptor for LPS, but induced less T cell proliferation than counterparts from healthy controls. In addition, in vitro treatment of PBMCs from BD-I patients with lithium for six days promoted the development of moDCs even in the absence of GM-CSF and IL-4. The authors demonstrated that the number of moDCs was increased in cultures from BD patients when compared to healthy controls [205]. The second study showed that after six days of treatment with lithium in vitro, moDCs exhibited decreased surface levels of CD14, but increased expression levels of CD1a, a lipid-presenting molecule, and enhanced capacity to induce proliferation of CD4^+^ T cells [206]. Especially the potential increase in lipid presentation efficiency warrants future investigation as the role of this unconventional and complex antigen class in inflammatory immune responses has only just begun to be revealed [207]. Taken together, these two studies suggest that lithium impacts maturation and the ability of DCs to induce adaptive immune responses and lipid recognition.

Drugs used in the treatment of AD have also been shown to mediate anti-inflammatory effects on murine DCs in vitro. Koh and colleagues showed that fluoxetine, a selective serotonin reuptake inhibitor (SSRI), inhibited LPS-induced TNF and IL-12p40 mRNA expression and protein secretion in bone marrow-derived DCs from IL-10 deficient mice by suppressing the kinase IKK within the NF-κB signaling pathway [208]. Similarly, another study showed that in vitro administration of desipramine, a norepinephrine reuptake inhibitor (NRI), reduced the secretion of TNF, IL-1β, and IL-12 by LPS-stimulated murine bone marrow-derived DCs [209]. As in humans, these results indicate that the anti-inflammatory and immunomodulatory effects of fluoxetine and desipramine can also be observed in murine DCs opening up the possibility to address the in vivo impact of these effects in animal models of depression-like behavior.

## 5. DCs in Rodent Models of Mood Disorders

Animal models of depression mirror certain aspects of the depressive syndrome, such as anhedonia, behavioral despair, and neurovegetative changes, and have significantly expanded our understanding of the pathogenesis of mood disorders. Depression-like behavior is induced by exposing rodents to acute and chronic stress paradigms, maternal separation, olfactory bulbectomy, selective breeding strategies for depression-related or resilient behavior, or by utilizing genetically modified animals. In addition, optogenetic and chemogenetic methods are used to investigate the neural circuit mechanisms within depression-like behavior [210,211]. Furthermore, a number of studies use experimental administration of endotoxins or pro-inflammatory cytokines to induce “sickness-behavior” in rodents and unravel the underlying molecular mechanisms of inflammation-induced depression. In social defeat models, a stress response is induced in defeated rodents, that elicits an inflammatory response and glucocorticoid resistance in immune cells [212,213,214,215].

Widely used tests to quantify behavioral despair or stress coping behavior in rodents are the forced swim test (FST) and the tail suspension test (TST). In both tests, animals are exposed to inescapable situations [216]. The sucrose/saccharin preference test (SPT) measures anhedonia and is based on the rodents’ natural preference for the sweetened solution [217,218,219]. Models of stress-induced behavioral change can lead to both depression- and anxiety-like behaviors, so experimentally induced anxiety-like behaviors are also commonly studied, e.g., through testing approach-avoidance conflicts. For further insights into animal models modelling depression, we recommend reviews on this topic [220,221,222,223]. In the following, we will highlight rodent models that have been used to better understand the effects of DCs in depression-like behavior.

### 5.1. Models of Inflammation-Induced Depression Induced by Endotoxin Administration

There is ample evidence that peripheral cytokines released in innate immunity trigger mood changes [12,33]. Following PAMP/DAMP recognition by innate immune cells, three cytokine-dependent pathways (humoral, neural, and cellular) mediate immune-brain communication. The “humoral” pathway relates to the entry of cytokines into the brain through “leaky” regions of the blood brain barrier such as circumventricular organs and the choroid plexus. The “neural route” involves activation of afferent nerve fibers that express cytokine receptors and relay signals to the brain, and the “cellular pathway” comprises the chemokine-dependent recruitment of immune cells to the brain during neuroinflammation [57,224]. DCs contribute to all three pathways due to (i) release of pro-inflammatory cytokines including IL-1β upon maturation and activation of afferent vagal nerve fibers expressing IL-1β receptors, (ii) their localization near afferent vagal nerve fibers and associated paraganglia, and (iii) their immigration into the brain during neuroinflammation, and local release of chemokines that recruit peripheral immune cells [48,160,225].

Peripheral administration of LPS in humans and rodents induces “sickness behavior” commonly used to study inflammation-related depression. In humans, sickness behavior is characterized by fatigue, social withdrawal, and decreased appetite. A few hours after injection, LPS induces anxiety symptoms and depressed mood [59]. These emotional changes correlate with fever and elevated serum IL-6 and TNF levels [59,226,227,228]. Rodents exposed to LPS show weight loss, decreased motor activity, and food intake, associated with increased proinflammatory cytokine levels in the periphery and brain. Subsequently, there is an increase in IDO levels, in neuroinflammation, and depression-like behavior [57,77,229,230,231]. Inflammation-induced depression also occurs after exposure of rodents to the viral mimetic poly I:C, which binds to TLR3 and the Rig-I-like receptor (RLR) MDA5 and has been associated with reduction in brain-derived neurotrophic factor (BDNF) signaling and increased levels of kynurenine [232]. Interestingly, endotoxins from Gram-positive bacteria (lipoteichoic acid; LTA) that bind to TLR2 can also induce neuroinflammation in mice, but without inducing behavioral changes [233]. One explanation could be that different signaling pathways are induced by these TLRs. Ligation of TLR2 and TLR4, activates NF-kB mediated signaling, which induces expression of genes for pro-inflammatory cytokines. In contrast, TLR3 and TLR4 induce a signaling pathway involving transcription factor IRF3 activation, which leads to production of type I IFN [234,235].

**Figure 1:**
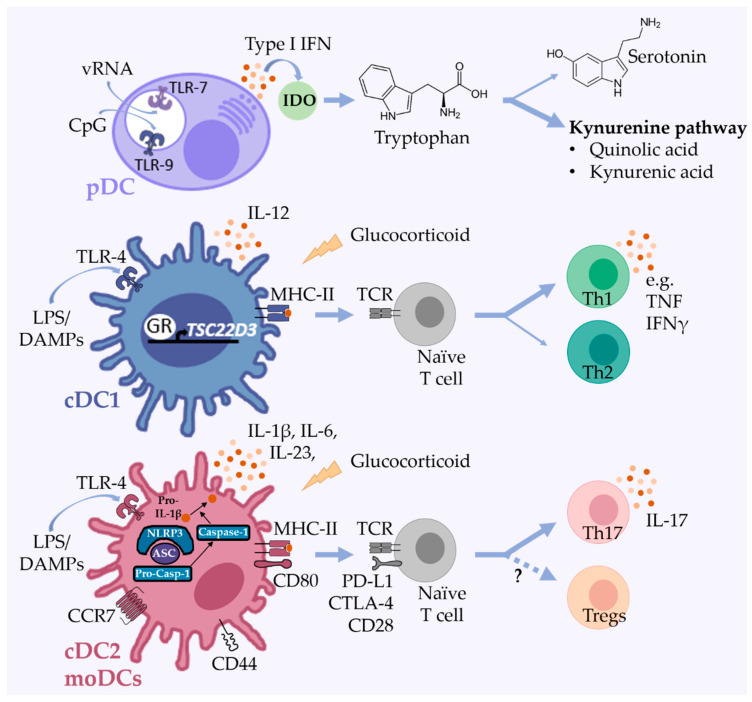
DC subsets in mood disorders and depression-like behavior. Plasmacytoid DCs (pDCs) produce high levels of type I IFN after sensing viral RNA by TLR-7 and/or CpG containing DNA by TLR-9 ligation. Type I IFNs induce IDO expression, leading to depletion of tryptophan, an important precursor of serotonin, and increased formation of neurotoxic kynurenine metabolites such as quinolic and kynurenic acid. Following social stress, glucocorticoids induce the expression of Tsc22 domain family protein 3 (TSC22D3) in DCs, a crucial glucocorticoid-dependent regulator of DC effector functions. Through secretion of IL-12 and presentation of antigens by MHC II, conventional DC1 (cDC1) induce Th1 cells that produce inflammatory cytokines involved in the development of AD, including TNF and IFNγ. Social stress leads to increased expression of CD80, CD44, and CCR7 in conventional DC2 (cDC2)/monocyte-derived DCs (moDCs) and the secretion of inflammatory cytokines, including IL-1β, IL-6, and IL-23 involved in Th17 cell development and maintenance. PAMPs (e.g., LPS) and DAMPs bind to PRRs and activate the pyrin domain-containing 3 (NLRP3) inflammasome complex leading to caspase-1 activation and maturation of e.g., IL-1β. IDO—indoleamine 2,3-dioxygenase; PAMPs—pathogen-associated molecular patterns; DAMPS—damage-associated molecular-patterns.

More specifically, LPS-binding to TLR4 activates a complex signaling pathway dependent on the adaptor molecules MyD88 and TRIF leading to translocation of NF-κB and IRF3 into the nucleus for transcription of inflammatory genes and type I IFN [55,77]. Although the exact mechanism of LPS-induced mood changes is still unclear, it has been proposed that TLR4/NF-κB signaling induces IDO concurrent with upregulation of inflammatory cytokines [77,236]. The enzyme IDO is expressed by several cell types such as fibroblasts, myeloid-derived suppressor cells, and myeloid cells including mature DCs [237,238,239]. IDO catalyzes the first and rate-limiting step of degradation of tryptophan, an important precursor of serotonin [240]. Increased IDO activity leads to impaired metabolism and depletion of tryptophan, increased formation of kynurenine, and accumulation of its toxic downstream metabolite quinolinic acid (Figure 1). This neurotoxic challenge is associated with depressive symptoms [57]. A large number of studies have demonstrated the association of depressive symptoms with type I IFN treatment [14,15,241]. Of note, a subset of human and murine DCs are highly capable to produce IDO after triggering IFN response elements in the IDO gene when exposed to type I and/or type II IFNs [240]. Two recent studies highlighted the role of IDO activity in type I IFN-induced depressive symptoms through induction of neurotoxic kynurenine metabolites. In patients affected by hepatitis C virus, IFNα treatment induced IDO expression and the increase of the neurotoxic quinolinic acid in the brain. Moreover, levels of quinolinic acid correlated with depressive symptoms [242]. Additionally, in another study, IFNα treatment increased depressive symptoms in patients with hepatitis C associated with an enhanced ratio of kynurenine/tryptophan, a correlate for IDO activity. The ratio of kynurenine/neuroprotective metabolite kynurenic acid was enhanced, thus reflecting enhanced neurotoxicity [243]. The causative involvement of IDO in depressive-like behavior was shown by blocking IDO with its antagonists 1-Methyltryptophan (1-MT), a treatment preventing LPS-induced depression-like behavior in mice [77]. Similarly, Hemmati and colleagues showed that exogenous application of GM-CSF mediated antidepressant effects in mice, likely by inhibiting TLR4/NF-κB-dependent induction of IDO [84].

With respect to BD, accumulating evidence points toward an impairment of the kynurenine pathway in BD. For example, enhanced IDO expression was found in anterior cingulate cortex of post-mortem brain tissues of BD patients [244]. A recent meta-analysis assessed kynurenine metabolites in peripheral blood in individuals with BD and HC. Interestingly, individuals with a manic episode showed the most pronounced reduction in peripheral blood levels of tryptophan, whereas kynurenic acid levels were more reduced among depressed subjects [245].

Although DCs were not examined in these studies, IDO expression by peripheral DCs may be of particular significance as a pathophysiological mechanism in mood disorders. IDO expressing DCs have been shown to exert tolerogenic effects and mediate suppression of effector T cells and promotion of Tregs [246]. On the other hand, pDCs are highly capable of secreting high levels of type I IFN and thus induce IDO activation in DCs [81,240]. Interestingly, inflammatory disorders associated with high levels of type I IFN such as lupus erythematosus, HIV/AIDS and rheumatoid arthritis are frequently associated with depressive symptoms [247,248]. Thus, DC-induced IDO activation, which can be triggered via type I IFN, may play an important role in MDD. Therefore, monitoring IDO-expressing DCs, tryptophan metabolism, and quinolinic acid levels in individuals with MDD or BD may lead to a better understanding of the role of DC-dependent immune activation and IDO in the development of mood disorders.

### 5.2. DCs in Animal Models of Stress-Induced Behavioral Changes

Changes in the phenotype and function of DCs in the stress response have been investigated in only few in vivo studies (Table 3). Nevertheless, findings from these experiments suggest important functions of DCs in the stress response and depression-like behavior that influence antiviral T-cell responses and tumor immunity [249,250,251]. Powell and colleagues examined the effect of a social stressor on DCs in the social disruption (SDR) paradigm in mice [252]. In SDR, rodents are defeated by an aggressive conspecific in their home cage [253]. Here, after six days of SDR, DCs from the spleen of subordinate animals expressed increased levels of MHC I, CD80, and CD44 compared to those from non-stress controls [252]. In addition, DCs from mice exposed to SDR secreted higher amounts of IL-6 and TNF after in vitro LPS stimulation and were glucocorticoid resistant. In a follow-up study, the authors demonstrated that adoptive transfer of DCs from stressed mice confers enhanced adaptive immunity to influenza A virus in recipient animals [250]. In summary, SDR induces glucocorticoid resistance and DC maturation associated with an enhanced capacity to induce antiviral T cell responses.

In a recent study on immune consequences of stress exposure, we compared DCs in stress-susceptible versus resilient mice exposed to chronic social defeat stress (SDS) [251]. Consistent with previous studies, a 10-day exposure to SDS induced social avoidance behavior (susceptibility) in approximately half of the animals, whereas the other animals showed social interaction (resilience) comparable to controls [257]. We found that DC frequencies were reduced in the spleens of all mice exposed to SDS, regardless of susceptibility or resilience. However, exclusively DCs from susceptible animals showed an enhanced maturation phenotype with increased expression of MHC II and co-stimulatory CD80 molecules. The T-cell differentiation cytokine IL-12 plays an important role in adaptive immune responses and has also been implicated in stress responses [258,259,260]. Interestingly, phenotypically mature DCs from susceptible mice did not show an increased capacity to produce IL-12. Instead, stress-resilient animals showed an increased proportion of IL-12-producing DCs after LPS stimulation. Thus, we defined a specific stress-related phenotype of DCs with phenotypically more mature DCs in susceptible mice versus an increased capacity of DCs to produce IL-12 in resilient animals [251]. Our findings of an altered phenotype of DCs in SDS were associated with higher blood levels of corticosterone (CORT) and increased numbers of Th17 cells in stress-susceptible mice compared to resilient mice or undefeated controls [251,254].

Glucocorticoids exert anti-inflammatory and immunosuppressive effects on several cell types including DCs. Following activation of the HPA axis during the stress response, elevated levels of the adrenal glucocorticoid CORT are produced [261]. Stimulation of bone marrow-derived DCs with CORT has been shown to impair LPS-induced up-regulation of maturation-associated markers. By binding to the glucocorticoid receptor (GR) on DCs, CORT inhibited transcription of CD80 and CD86, induced intracellular retention of MHC II, and impaired LPS-induced production of IL-6, IL-12, and TNF. Moreover in vivo, treatment of mice with CORT reduced their ability to prime naïve CD8^+^ T cells [262]. Another study investigated the effect of CORT on the ability of DCs to process and present virally expressed antigens to CD8^+^ T cells. CORT suppressed the formation of peptide-MHC I complexes on the surface of virus-infected DCs and decreased their T cell stimulation capacity. Of note, DCs alter expression of GR isoforms that control sensitivity to glucocorticoids depending on their maturation stage. Thus, only mature, but not immature DCs are sensitive to glucocorticoid-induced apoptosis after in vivo and in vitro glucocorticoid stimulation due to expression of proapoptotic GR isoforms [263]. Interestingly, DCs may therefore become resistant to the suppressive effects of CORT after chronic social stress [252]. This may explain why these indirect immunosuppressive effects of social defeat can affect DCs in different ways depending on their maturation stage.

A recent study defined impressively that changes in DC function represent a mechanistic connection between social stress and reduced response to immunogenic chemotherapy [249]. The authors showed that glucocorticoid-dependent regulation of DC effector functions in social stress depends on TSC22D3 (Tsc22 domain family protein 3), also known as glucocorticoid-induced leucine zipper (GILZ) protein. TSC22D3 has previously been shown to mediate many glucocorticoid effects in immune and non-immune cells and to induce an anti-inflammatory phenotype in myeloid cells. TSC22D3 also regulates antigen processing and presentation by DCs and thus mediates most glucocorticoid effects in both tolerogenic and immunogenic DCs [264,265]. Yang and coauthors showed in a murine tumor model that SDS upregulated the expression of TSC22D3 in tumor-infiltrating DCs which was dependent on GR signaling [249]. TSC22D3 reduced the ability of DC to produce type I IFN and induce IFN-γ secretion in tumor-infiltrating T cells. Importantly, TSC22D3 mediated immunosuppression and abolished the efficacy of immunogenic chemotherapy and suppressed cancer-preventive immunity strategies. In addition, a correlation between plasma CORT levels and TSC22D3 expression in PBMCs has been found in patients with cancer and negative mood [249]. These important findings shed light on the relevance of DCs in psychosocial stress responses in antitumor immunity.

Another gene involved in social stress-dependent regulation of DC functions is *DNMT1* coding for the DNA methyltransferase 1, a key regulator of DNA methylation [266]. In a recent study it was shown that SDS in mice increased CORT plasma levels, and induced downregulation of DNMT1 and upregulated CCR7 expression in skin DCs. At a functional level, social stress exacerbated experimentally-induced atopic dermatitis in these animals [255].

Recently, the effects of repeated social defeat (RSD) with social disruption (SDR) on DCs of the spleen and peripheral blood were compared in mice [256]. Both stress paradigms equally induced social avoidance in the same manner and caused a decrease in the proportion of DC subsets (cDC1 and cDC2) in the bone marrow of defeated animals. However, DCs in the peripheral blood of the subordinate animals were reduced only when the animals were subjected to SDR but not RSD. These findings suggest that RSD alters the distribution of DCs and possibly migration to other sites such as the brain.

## 6. Future Perspectives

**Figure 2:**
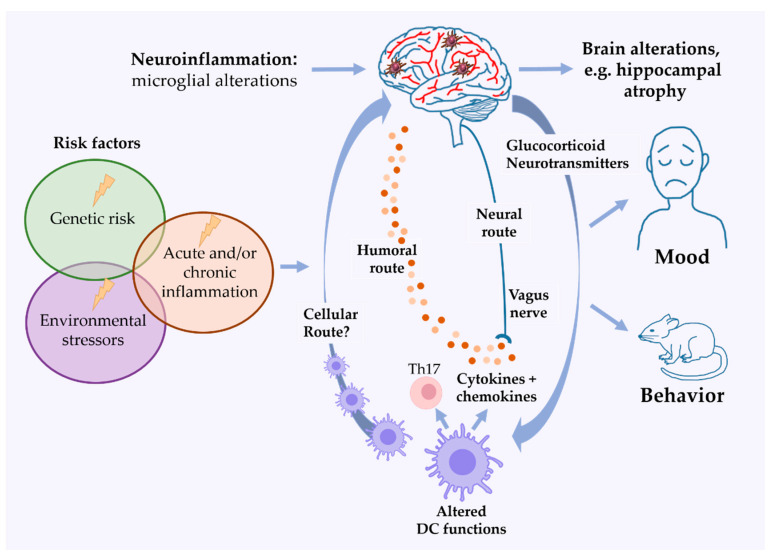
Dendritic cells in peripheral immune responses and neuroinflammation in mood disorders. A complex interplay of genetic and environmental factors and/or chronic inflammation can lead to altered functions in immune cells, including dendritic cells (DCs). DCs can induce neuroinflammation in many ways, e.g., by secretion of inflammatory cytokines and/or induction of Th cells, such as Th17 cells (see Figure 1). Peripheral DCs may modulate neuroinflammation by invasion into the brain (cellular route) and secretion of cytokines and chemokines that reach the brain (humoral route) and activate afferent nerve fibers (neural route). Microglial activation has been found in AD and in corresponding rodent models and may lead to brain alterations such as hippocampal atrophy, a feature of AD.

DCs are able to shape the immune response in stress and mood disorders in several ways. They are sensors of DAMPs and can induce sterile inflammation in innate immunity on the one hand and trigger adaptive T-cell responses on the other (Figure 2). Both arms of the immune response have been seen altered in AD. In this review, we summarized the distinctive key findings on DCs and their effector molecules in individuals with AD, in clinical trials and in vitro studies, and in mouse models for depression-associated behavior. The capacity of DCs to migrate, secrete pro- and anti-inflammatory cytokines and chemokines, and activate T-cell responses, as well as the ability for the large-scale ex vivo generation and gene modification of DCs from human blood monocytes make them ideal candidates for therapeutic applications in AD. Promising studies link the phenotype and function of DCs to stress resilience and suggest an essential role for these cells in controlling the efficacy of tumor therapy after stress. However, large parts of DC biology in AD still remain to be elucidated, precluding definitive conclusions. There are many unanswered questions, such as the influence of DC subtypes on neuroinflammation and behavior, the specific immunosuppressive effects of stressors on DCs in different maturation stages, and their functional impact on the development and progression of AD in humans. A better understanding of the potentially multifaceted roles of DCs in the stress response with relevance to AD may point to novel treatment strategies by employing this cell type as therapeutic targets in mood disorders.

## Figures and Tables

**Table 2:** Chemokines and chemokine receptors involved in AD and depression like behavior.

**Chemokine Receptor**	**Ligand**	**Function in DCs**	**Impact on Behavior**	**Reference**
CCR4	CCL17, CCL22	Multiple functions including migration and secretion of GM-CSF and IL-23	CCR4 knockout mice show reduced locomotor activity, less anxiety-related behavior, and diminished social exploration	[137]
CCR6	CCL20	Chemotaxis of DCs to inflammatory sites and the brain	CCR6 knockout mice show higher locomotor activity, lower anxiety, and reduced preference for saccharin (in weekly testing)	[136]
CCR7	CCL19, CCL21	Migration, differentiation, endocytosis, release of cytokines	CCR7 knockout mice show impaired learning (Barnes maze), higher anxiety, and reduced preference for saccharin (in weekly testing)	[136]
CX3CR1	CX3CL1	Induces e.g., actin polymerization and migration of DCs, independent of their maturation status	CX3CR1 knockout mice show increased resilience to stress-induced depression-like behavior	[138,139]
**Ligand**	**Chemokine Receptor**	**Function in DCs**	**Clinical Studies**	**Reference**
CCL2	CCR2	Migration, maturation, and production of IL-12	Increased CCL2 serum levels in patients with affective disorders	[140]
CXCL8	CXCR1, CXCR2	Chemotaxis of immature DCs to inflammatory sites	Increased CXCL8 blood levels in depressed individuals	[141]
CXCL12	CXCR4	Migration of DCs from the skin into the regional lymph nodes	Reduced CXCL12 plasma levels in patients with non-affective psychosis	[142]

**Table 3:** DCs in rodent models of AD.

Animal Model	Duration	Tissues Analyzed	Alterations Found in DCs	Reference
SDR	6 days	Spleen	Increased MHC I, CD80 and CD44 expression and glucocorticoid resistance ex vivo and IL-6 and TNF productionafter in vitro stimulation with LPS	[252]
SDR	6 days	Spleen, lung	Enhanced maturation and capacity to induce antiviral T cell responses, adoptive transfer of splenic DCs from SDR exposed mice confers immunity towards influenza A virus, glucocorticoid resistance	[250]
SDS	10 days	Spleen	Increased MHC II and CD80 expression by DCs of susceptible mice, higher IL-12^+^ DC proportions in resilient mice	[251,254]
SDS	10 days	Spleen, LN, tumor	Upregulated TSC22D3 expression and reduced capability to produce type I IFN in tumor-infiltrating DCs after SDS and reduced capability to induce IFN-γ secretion in tumor-infiltrating T cells	[249]
SDS	10 days	Skin	Downregulated *DNMT1* and upregulated CCR7 expression in skin DCs, exacerbated experimentally-induced atopic dermatitis	[255]
SDR and SDS	6 (SDR) and 10 (SDS) days	Spleen, blood, bone marrow	Reduced cDC1 and cDC2 cell percentages in bone marrow after SDR and SDS; reduced DC percentages in peripheral blood of subordinate animals after SDR	[256]

SDR—social disruption; SDS—chronic social defeat stress; LN—lymph node; DCs—dendritic cells; cDC1—conventional dendritic cells 1; cDC2—conventional dendritic cells 2; CORT—corticosterone; TSC22D3—TSC22 domain family member 3; *DNMT1*—DNA Methyltransferase 1.

## Data Availability

Not applicable.
